# W-Band GaAs pHEMT Power Amplifier MMIC Stabilized Using Network Determinant Function

**DOI:** 10.3390/mi16010081

**Published:** 2025-01-12

**Authors:** Seong-Hee Han, Dong-Wook Kim

**Affiliations:** Department of Radio and Information Communications Engineering, Chungnam National University, 99 Daehak-ro, Yuseong-gu, Daejeon 34134, Republic of Korea; hee.hans@o.cnu.ac.kr

**Keywords:** GaAs pHEMT, W-band, power amplifier, MMIC, network determinant function

## Abstract

This paper presents a W-band power amplifier monolithic microwave integrated circuit (MMIC) that is designed for high-precision millimeter-wave systems and fabricated using a 0.1 µm GaAs pHEMT process. The amplifier’s stability was evaluated using the network determinant function, ensuring robust performance under both linear and nonlinear conditions. Simultaneous matching for gain and output power was achieved with minimal passive elements. The developed power amplifier MMIC exhibits a linear gain exceeding 20 dB and an input return loss greater than 6 dB across the 88–98 GHz range. It delivers an output power of 23.8–24.1 dBm with a power gain of 17.3–17.9 dB in the 88–97 GHz range and achieves a maximum power-added efficiency (PAE) of 24% at 94 GHz.

## 1. Introduction

The W-band frequency range (75–110 GHz) is widely used in high-precision systems such as short-range imaging, remote sensing, and automotive radar systems [1,2,3,4,5]. Developing MMICs for millimeter-wave applications presents significant challenges, particularly in achieving a low noise figure (NF) and high output power, both of which are critical for enhancing the signal-to-noise ratio (SNR) and system compactness.

Among various technologies, GaAs pseudomorphic high electron mobility transistors (pHEMTs), GaAs metamorphic high electron mobility transistors (mHEMTs), and GaN HEMTs offer competitive performance for W-band applications. While GaN HEMTs excel in achieving high saturated output power, their cost-to-power ratio remains less favorable compared to the 0.1 μm GaAs technology [6]. Consequently, GaAs HEMTs present a viable alternative, offering advantages such as high electron mobility, low turn-on voltage, minimal leakage current, and high power-added efficiency (PAE) [7,8,9].

In multi-stage power amplifiers, the output stage transistor’s drain port must be matched to its optimal load impedance to maximize output power, while the transistors in preceding driving stages require conjugate matching of their S_22_ parameters for sufficient linear gain. This study focuses on the design and fabrication of a four-stage, single-ended power amplifier operating at 94 GHz using the 0.1 µm GaAs pHEMT process. The amplifier is intended for both military and civilian applications [10,11,12]. Stability under both small- and large-signal conditions is ensured using the network determinant function (NDF) and the Rollett stability factor, K [13].

The conventional K-factor stability criterion often falls short for multi-stage circuits, as it does not account for all oscillatory conditions, especially under nonlinear operation. Platzker et al. highlighted the need for evaluating the network determinant’s zeros in the right-half plane (RHP) as a prerequisite for applying the K-factor test [14]. This study incorporates the NDF to address stability issues comprehensively, ensuring reliable operation in both linear and nonlinear regimes.

## 2. Circuit Design

### 2.1. 0.1 μm GaAs pHEMT Process

This study employs a 0.1 µm GaAs pHEMT process from Win Semiconductors to design a four-stage power amplifier. The design utilizes Keysight’s Advanced Design System (ADS) for circuit modeling and electromagnetic simulations. Passive elements, including microstrip lines and stubs, are simulated using the 2.5-D momentum electromagnetic tool in ADS.

**Figure 1:**
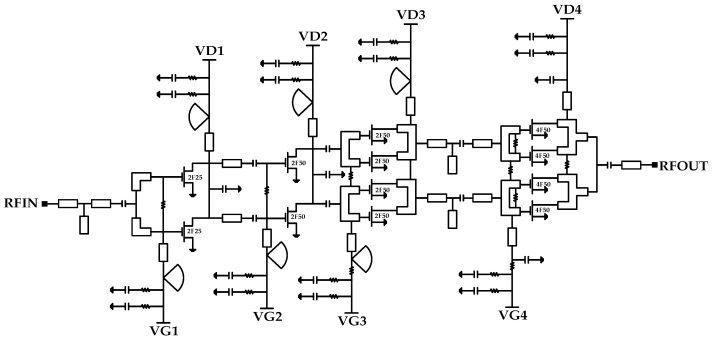
Circuit schematic of the W-band power amplifier.

The first three driving amplifier stages utilize transistors with gate widths of 2 × 25 μm and 2 × 50 μm. The final stage comprises four 4 × 50 μm transistors in parallel to enhance its output power. Figure 1 shows the circuit schematic diagram.

RF choke inductors are implemented using radial stubs and λ/4 microstrip lines, and RC traps are added in the biasing networks to mitigate potential oscillation issues. Transistors are biased for Class A operation (V_DS_ = 4 V, I_DS_ = −0.4 V) to achieve adequate linearity and gain.

As shown in Figure 1, T- and L-section low-pass impedance matching circuits, using low-impedance microstrip lines and small-valued shunt capacitors, are designed to maintain their impedance trajectories within the low-Q circle region on the Smith chart.

**Figure 2:**
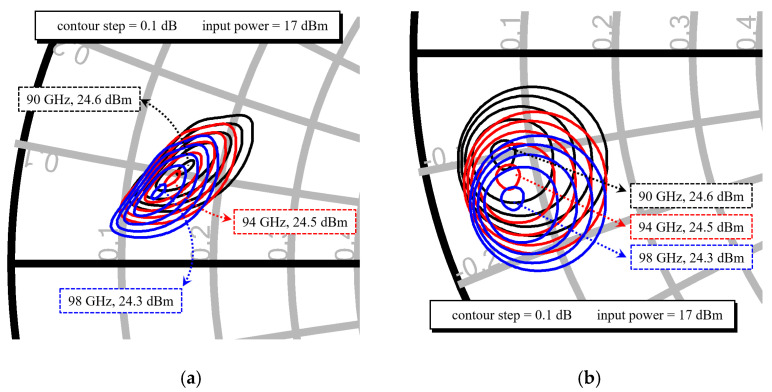
Simulated source pull and load pull results of the 4 × 50 μm transistor: (**a**) load pull; (**b**) source pull.

The source-pull and load-pull simulation results for the 4 × 50 μm transistor employed in the final stage are presented in Figure 2. These simulations are conducted at an input power level of 17 dBm across the frequency range of 90–98 GHz, with a circle trajectory step of 0.1 dB. The 4 × 50 μm transistor demonstrates a predicted saturated output power of 21 dBm. Accounting for a power margin of 1.5 dB and a matching loss of 1 dB, the conservatively estimated output power of the power amplifier is approximately 24.5 dBm.

The first and second stages are conjugately matched using S-parameters to achieve high gain, while the third and final stages are matched to the transistor’s optimal output impedance to maximize the output power. To minimize the inherent losses in the matching circuits, distributed matching sub-circuits employing microstrip elements are used for inter-stage impedance matching.

In the design frequency range, interdigitated capacitors are used to achieve precise sub-pF capacitance values, avoiding the higher tolerances associated with metal-insulator-metal (MIM) capacitors. Additionally, thin film resistors (TFRs) are integrated into the gate biasing networks to deliver stable gate voltages and effectively suppress odd-mode oscillations. Compact and efficient drain biasing is achieved using long microstrip lines with shunt MIM capacitors grounded at the midpoints of the microstrip lines.

### 2.2. Network Determinant Function

The K-factor is derived from the S parameters of a two-port circuit, as defined by Equations (1) and (2). Circuit stability is typically determined based on whether the K-value exceeds 1. However, it is important that even if the K-value is less than 1, the circuit may still operate stably under certain conditions [13].

For multi-stage circuits, relying only on the K-value as a stability criterion can lead to misleading conclusions, particularly due to the impact of matching losses between stages. In such cases, the normalized determinant function (NDF) provides a more robust approach to analyzing the circuit stability. The NDF evaluates both linear and nonlinear stability by applying a perturbation current to potentially unstable nodes, such as gate and drain nodes of transistors, and identifying oscillation frequencies using the determinant function. This is especially effective for identifying and mitigating stability issues in complex multi-stage circuit design [14,15].

(1) $$K=\frac{1-{\left|S_{11}\right|}^{2}-{\left|S_{22}\right|}^{2}+{\left|\Delta \right|}^{2}}{2\left|S_{21}S_{12}\right|}$$

(2) $$\Delta =S_{11}S_{22}-S_{12}S_{21}$$

The NDF is derived using Equation (3), where |Y(ω)| represents the admittance matrix at the suspected node, and |Y₀(ω)| corresponds to the admittance matrix when all active elements in the circuit are passively transformed. In this context, passive operation of a transistor implies that its transconductance is non-positive, ensuring that the transistor cannot induce oscillation. The NDF for the entire circuit is determined by multiplying the individual NDF values calculated for each suspected node. Passive operation at the gate node is achieved when the gate is biased below the pinch-off voltage, while at the drain node, passive operation occurs when the drain bias voltage is set to 0 V [16].

(3) $$NDF\left(\omega \right)=\frac{\left|Y\left(\omega \right)\right|}{\left|Y_{0}\left(\omega \right)\right|}$$

(4) $$Enciclements=\frac{unwrap(∠NDF)}{unwrap(∠NDF{｜}_{\omega =0})\times (-360)}$$

**Figure 3:**
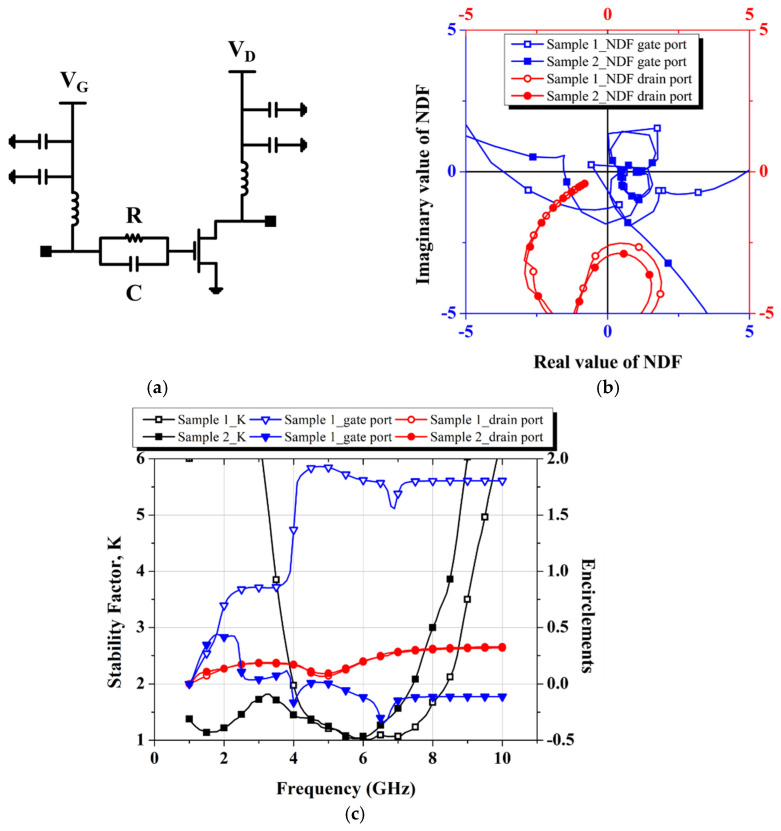
Stability check using K and NDF: (**a**) Test sample sub-circuit; (**b**) NDF graphs; (**c**) Encirclements (sample circuit 1: R = 50 Ω, C = 1 pF, sample circuit 2: R = 50 Ω, C = 6.8 pF).

The NDF plot is presented in Figure 3, showing the results from test sample circuits used for comparison between the NDF and K-factor. Sample circuits 1 and 2 include the sub-circuit shown in Figure 3a, with the only difference being the resistor and capacitor values at the transistor gate. When the NDF is plotted as a function of frequency on a polar chart, if the curve encircles the origin more than once in a clockwise direction, the test circuit is identified as unstable [14], as demonstrated in Figure 3b.

Figure 3c numerically represents the number of times the NDF curve encircles the origin on the polar chart as the frequency increases, derived from Equation (4). According to this plot, both sample circuits satisfy K > 1 across the full band, but the NDF of the test sample circuit 1 encircles the origin more than once above 4 GHz, indicating instability. This emphasizes the importance of first performing an NDF check and adjusting the passive element values before estimating the stability using the K-factor.

**Figure 4:**
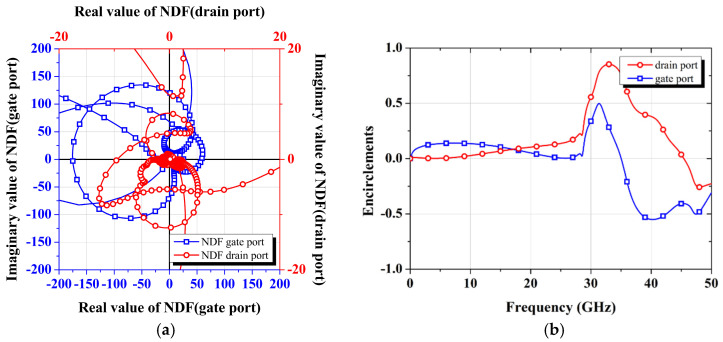
NDF graphs of the designed W-band power amplifier MMIC: (**a**) polar chart; (**b**) encirclements.

In the development of the W-band power amplifier, the stability criterion described above was applied to avoid the potential oscillation issues. Figure 4 displays the NDF waveform for the designed power amplifier, confirming stable operation as no RHP zeros are present across the entire frequency range.

Before assessing the oscillation (unstable condition) during nonlinear (or large-signal) operation, the circuit must first be unconditionally stable during linear (or small-signal) operation. In other words, nonlinear NDF calculations are only performed after the circuit passes the linear NDF stability test. In this context, $\left|Y\left(\omega \right)\right|$ in Equation (3) represents the admittance matrix at the suspected node under large-signal input conditions, while $\left|Y_{0}\left(\omega \right)\right|$ refers to the admittance matrix when the small-signal power is applied. By following this methodology, the circuit stability can be evaluated for each combination of the input power and frequency [17].

**Figure 5:**
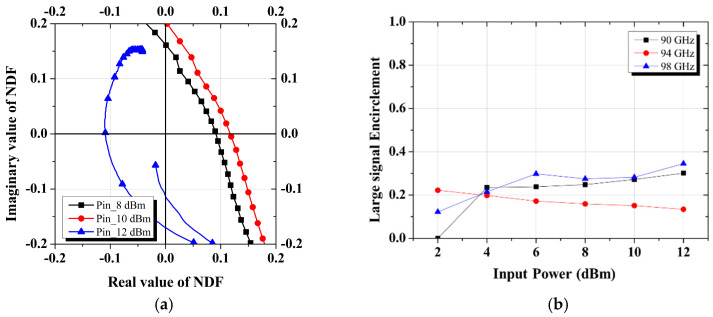
NDF graphs of the designed W-band power amplifier MMIC during nonlinear operation: (**a**) polar chart at 94 GHz; (**b**) encirclements.

Figure 5 presents the results of applying the nonlinear NDF test to the designed W-band power amplifier MMIC shown in Figure 1. Figure 5a illustrates the NDF trajectories at 94 GHz with the input power. Since the trajectory does not encircle the origin, it can be inferred that there are no RHP zeros, indicating that the circuit is stable. Additionally, the graph shown in Figure 5b depicts the large-signal encirclement as the input power and operating frequency change and shows that no large-signal encirclement exceeding 1 is observed during the nonlinear operation [14,17]. Therefore, it is concluded that the designed power amplifier remains stable during the nonlinear operation.

## 3. Measurement

**Figure 6:**
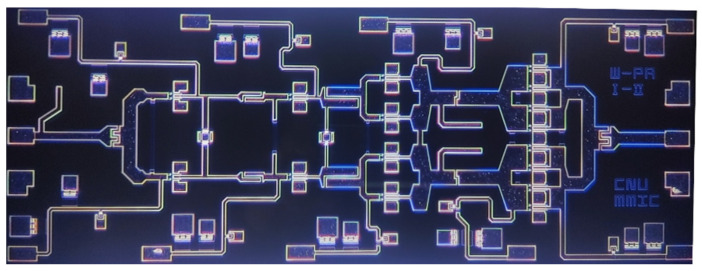
Photograph of the fabricated W-band power amplifier MMIC.

Figure 6 shows the fabricated W-band power amplifier MMIC, which occupies an area of 1.0 × 2.6 mm^2^. The MMIC chip is mounted onto a CuW carrier using epoxy bonding. For the bias voltage supply, single-layer capacitors (SLCs) with the values of 10 nF and 100 pF are placed between DC pads on the chip and the evaluation PCB. The S-parameter measurements in the W-band are performed using an on-wafer probe system, a vector network analyzer (Keysight N5234B), WR-10 frequency extension modules (Keysight WR10-VNAX), and mm-wave test controllers (Keysight N5295A).

To measure the large-signal performance in the W-band, Ku-band signals are up-converted to W-band signals using a 6× frequency multiplier. The output of the multiplier is transferred to a W-band driving amplifier and a W-band attenuator to control the input power. The measurement setup includes a signal generator (Keysight E8257D), a 6× multiplier (Millitech AMC-10-RNHB3), power sensors (Anritsu MA24510A), and a driving amplifier (Eravant SBP-9039832526-1010-E1).

**Figure 7:**
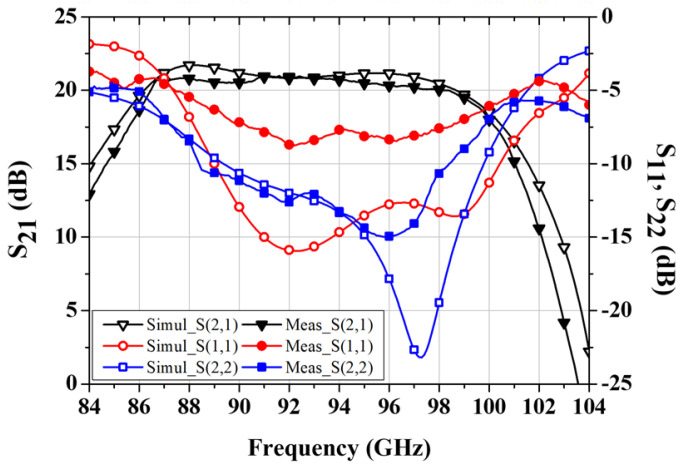
Measured S-parameter results of the W-band power amplifier MMIC.

Figure 7 shows the measured S-parameter results of the power amplifier MMIC under the bias conditions of V_DS_ = 4 V and I_DQ_ = 360 mA. The input return loss exceeds 6 dB in the 88–98 GHz frequency range, and the gain is greater than 20 dB. The gain is decreased by about 1 dB, and input return loss is degraded by about 5 dB, partly due to some mismatch in the W-band between the transistor model and the transistor’s measured data, while the measured output return loss is in good agreement with the simulation data.

**Figure 8:**
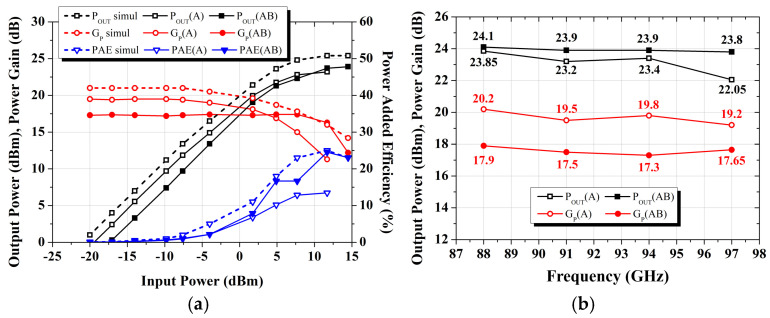
Large-signal measurement results of the W-band power amplifier MMIC: (**a**) Output power performance with input power at 94 GHz; (**b**) Saturated output power and power gain in the 88–97 GHz region.

Figure 8a presents large signal characteristics of the fabricated W-band power amplifier MMIC at 94 GHz. The measurement under Class A bias conditions shows slight degradation in the output power compared to the simulated results. To enhance the output power, the bias conditions of the final stage were changed to a Class AB mode. As a result, the gain slightly decreased to 17.3–17.9 dB, but the output power increased by a maximum of 1.75 dB. Additionally, the maximum power-added efficiency (PAE) significantly improved from 13% to about 24%. Figure 8b shows the saturated output power and power gain as functions of frequency. Under Class-A bias conditions (gain mode), the output power was measured to range from 22.05 to 23.85 dBm, with a power gain of 19.2 to 20.2 dB. In contrast, under the Class-AB bias condition of the final stage (power mode), the output power increased to 23.8–24.1 dBm, while the power gain decreased to 17.3–17.9 dB within the 88–97 GHz frequency range.

Table 1 compares the results of this work with previously published W-band power amplifiers [18,19,20,21,22]. The fabricated W-band power amplifier MMIC in this work demonstrates comparable gain and output power performance to other published results while occupying a smaller area.

## 4. Conclusions

This study presents a compact and efficient W-band power amplifier MMIC using a 0.1 μm GaAs pHEMT process. To ensure both linear and nonlinear circuit stability, the normalized determinant function was employed under both small- and large-signal conditions across the frequency range from DC to 110 GHz. The developed amplifier achieves a linear gain of over 20 dB within the 88–98 GHz range. Under the large-signal operation, it delivers a saturated output power of 23.8–24.1 dBm with a power gain of 17.3–17.9 dB in a continuous wave mode. When the final amplification stage operates in a Class A bias mode, the power amplifier demonstrates an output power of 22.1–23.9 dBm with a power gain of 19.2–20.2 dB. With the final stage operating in a Class AB mode, the output power increases by a maximum of 1.75 dB, and the PAE improves by 11%, with a maximum gain reduction of 2.5 dB.

The W-band power amplifier developed in this study offers high output power while maintaining a compact size, making it suitable for a wide range of applications, including high-resolution radar systems and proximity fuses for both military and civilian purposes.

## Figures and Tables

**Table 1:** Comparison of this work with previously published W-band power amplifier MMICs using the 0.1 μm GaAs process.

Work	Frequency (GHz)	Linear Gain (dB)	Output Power (dBm)	DC Power (mW)	PAE (%)	Chip Size (mm^2^)
[18]	84–103	15	21.5	1260	N.A.	2 mm^2^
[19]	75–84	25	20	500	10.5 *	2.1 mm^2^
[20]	75–110	16.5	15.5	355	9.6	5 mm^2^
[21]	80–100	12	24.5	350	N.A.	3.5 mm^2^
[22]	80–98	≥20	20	158	13.3	3.72 mm^2^
This work	88–97	≥20	22.1–23.9 (gain mode)	1440	13	2.6 mm^2^
			23.8–24.1 (power mode)	1010	24	

*: estimated value.

## Data Availability

Data are contained within this article.
